# Retraction: Tuning the chemistry of graphene oxides by a sonochemical approach: application of adsorption properties

**DOI:** 10.1039/d0ra90040k

**Published:** 2020-04-23

**Authors:** Yubing Sun, Shubin Yang, Congcong Ding, Zhongxiu Jin, Wencai Cheng

**Affiliations:** Institute of Plasma Physics, Chinese Academy of Science P.O. Box 1126 Hefei 230031 P. R. China sunyb@ipp.ac.cn +86 551 65591310 +86 551 65592788; School of Environment and Chemical Engineering, North China Electric Power University Beijing 102206 P. R. China; School for Radiological and Interdisciplinary Sciences, Soochow University 215123 Suzhou P. R. China; Collaborative Innovation Center of Radiation Medicine of Jiangsu Higher Education Institutions P. R. China; Faculty of Engineering, King Abdulaziz University Jeddah 21589 Saudi Arabia

## Abstract

Retraction of ‘Tuning the chemistry of graphene oxides by a sonochemical approach: application of adsorption properties’ by Yubing Sun *et al.*, *RSC Adv.*, 2015, **5**, 24886–24892, DOI: 10.1039/C5RA02021B.

The Royal Society of Chemistry, with the agreement of the named authors, hereby wholly retracts this *RSC Advances* article due to concerns with the reliability of the data in the published article.

The TEM image in Fig. 1B duplicates data published in another publication by Pan *et al.*, but presented as different materials.^1^

The AFM images in Fig. 1C and D illustrate duplication of data, given that these experiments were reported under different reaction conditions.

The EXAFS spectra in Fig. 4 duplicate data in another publication, but reported as different materials.^2^

Given the number and significance of the concerns about the validity of the data, the findings presented in this paper are no longer reliable.

Signed: Yubing Sun, Shubin Yang, Congcong Ding and Wencai Cheng

Date: 27^th^ March 2020

Zhongxiu Jin was contacted but did not respond.

Retraction endorsed by Laura Fisher, Executive Editor, *RSC Advances*

## Supplementary Material
